# Review of State-of-the-Art Degradation Models for Lithium-Ion Batteries

**DOI:** 10.3390/e28060669

**Published:** 2026-06-11

**Authors:** Richa Vinod Tiwari, Lakshmana C. Rao, Cemal Basaran

**Affiliations:** 1 Department of Applied Mechanics & Biomedical Engineering, Indian Institute of Technology Madras, Chennai 600036, India; am22d035@smail.iitm.ac.in (R.V.T.); lakshman@zmail.iitm.ac.in (L.C.R.); 2 Department of Civil, Structural and Environmental Engineering, University at Buffalo, New York, NY 14260, USA

**Keywords:** battery, degradation, sustainability, modeling

## Abstract

Lithium-ion batteries (LIBs) are widely used across a range of applications; however, they degrade over time due to various factors, including repeated charge–discharge cycling, material aging, and environmental conditions. Degradation models play a crucial role in predicting the lifespan of LIBs and in optimizing their design and operational strategies. This paper presents a comprehensive review of state-of-the-art degradation models for LIBs. The reviewed models primarily address key degradation mechanisms, including solid electrolyte interphase (SEI) formation, lithium plating, and particle fracture. For each mechanism, the underlying modeling approaches, their development, advantages, limitations, and associated challenges are critically discussed. Finally, this review identifies existing gaps in battery degradation modeling and proposes the Unified Mechanics Theory (UMT), which is the unification of laws of Newton and the second law of thermodynamics, and uses entropy as a degradation metric, as a promising alternative framework for capturing the coupled and multifaceted nature of battery degradation processes.

## 1. Introduction

Lithium-ion batteries are integral to the global transition toward sustainable energy systems. This shift is accelerated by international climate mandates, such as the United Nations Framework Convention on Climate Change (UNFCCC) and reinforced by the International Energy Agency (IEA) reports showing that countries representing the majority of global carbon dioxide emissions are now prioritizing cleaner energy alternatives [1,2]. Within the transportation sector, electric vehicles (EVs) offer higher energy efficiency than internal combustion engine (ICE) vehicles, with reported efficiency gains of up to 30%, even when electricity generation involves fossil fuels [3]. EVs rely almost exclusively on lithium-ion batteries for onboard energy storage. Although this technology is mature in consumer electronics, large-scale transportation applications introduce additional challenges related to cost, charging time, driving range, recycling, and supporting infrastructure [4]. Safety remains a critical concern, as degradation-driven thermal instability can result in fires or explosions [5]. Addressing these risks requires a mechanistic understanding of battery degradation processes and the development of predictive strategies for battery health assessment [4].

Degradation in lithium-ion batteries refers to irreversible electrochemical, thermal, and mechanical processes that progressively reduce capacity, power capability, and structural integrity [6]. Under severe operating conditions, degradation-induced heat generation, lithium accumulation, and stress evolution can interact, promoting thermal instability. In electric vehicle applications, such degradation directly constrains battery lifetime, safety, and cost, making reliable prediction of degradation progression and failure onset a central technical challenge [7].

Accurate prediction of battery degradation requires physics-based mathematical descriptions capable of resolving the dominant degradation mechanisms and their coupled multi-physics interactions across relevant length and time scales. However, many existing monitoring and control approaches rely on empirical correlations with limited validity beyond their calibrated operating conditions [8]. Physics-based degradation-focused modeling frameworks provide mechanistic insight into failure pathways, enable identification of critical operating regimes, and support strategies for lifetime extension and safety enhancement [9,10].

While battery aging mechanisms have been extensively summarized in previous literature, existing reviews predominantly focus on describing simple battery chemistry or detailing the physical phenomena of single degradation mechanisms in isolation. This review distinguishes itself by explicitly shifting the focus away from basic battery chemistry to provide a critical and comprehensive analysis of degradation models and their underlying mathematical formulations. To address the gaps in current literature, this review seeks to answer the following key questions: (1) What are the underlying mathematical structures and limitations of current physics-based degradation models for the three primary degradation mechanisms? (2) What are the fundamental limitations of using mechanism-specific parameters to predict long-term battery performance? and (3) How can entropy-based formulations provide a thermodynamically consistent and unified alternative to existing fragmented modeling approaches?

To answer these questions, this paper is organized as follows. First, a brief overview of electrochemical cell operation, including the fundamental governing equations, is provided to establish the foundation for the subsequent mathematical analysis. Next, we systematically explain the underlying physical mechanisms of the three dominant degradation modes (SEI formation, lithium plating, and particle fracture) and then evaluate their state-of-the-art modeling approaches, identifying key mathematical gaps in current research. Following this critical assessment, entropy-based degradation descriptions are introduced. Finally, the review highlights the potential of physics-based formulations grounded in Unified Mechanics Theory, without reliance on empirical curve fitting, as a promising direction for advancing the understanding and prediction of battery degradation.

## 2. Electrochemical Cells: A Detailed Explanation

A lithium-ion battery pack is a structure consisting of modules and cells. An electrochemical cell is the fundamental unit of a battery, and it consists of two porous electrodes (positive and negative) with a porous separator in between, all three sandwiched between a pair of metallic current collectors. The electrodes are made up of porous microscopic particles of active materials, which are connected by a polymer binder doped with conducting material like carbon. The mixture of active material and doped polymer binder is then spread on current collectors made of conducting materials like aluminum and copper [11].

**Figure 1:**
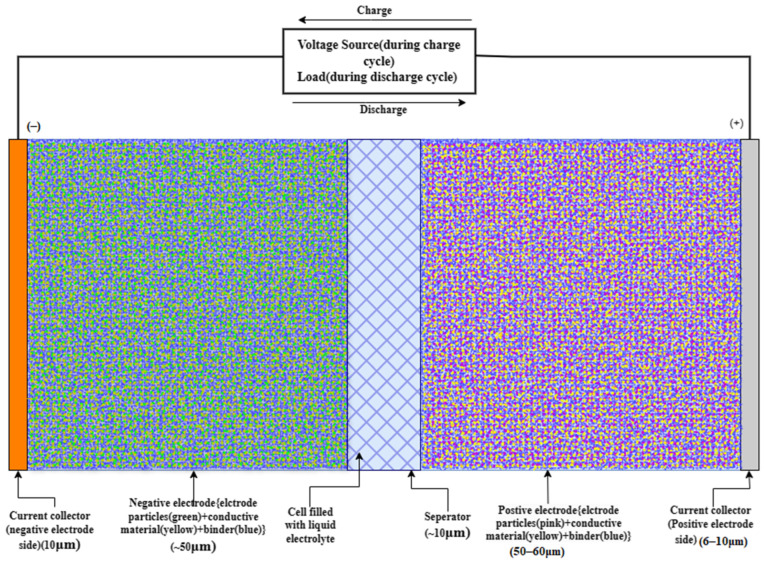
Schematic diagram of an electrochemical cell [11,12].

A separator is a semi-permeable, non-conductive membrane placed between the negative and positive electrodes to prevent direct electrical contact. The complete structure is then flooded with an ionically conducting and electrically insulating electrolyte (like Ethylene carbonate (EC), Dimethyl carbonate (DMC), propylene carbonate (PC), etc.). The setup is then placed in a casing made of stainless steel [11]. A schematic diagram of a lithium-ion battery and its microstructure is shown in Figure 1. On the left is the negative electrode, generally made up of graphite with a small addition of silicon particles to enhance capacity and conductivity. On the right side is the positive electrode, made up of materials such as lithium cobalt oxide, lithium iron phosphate, and nickel–manganese–cobalt (NMC) oxides, etc.

A charge cycle is one in which lithium ions from the positive electrode migrate to the negative electrode via the electrolyte and are stored in the interlayer spaces of graphite. During a discharge cycle, a load is connected to the external circuit. Due to the thermodynamic instability of lithium ions and electrons in the lithiated state, they migrate back to the positive electrode. Electrons return to the positive electrode through the external circuit, while lithium ions migrate back through the electrolyte. One charge process followed by one discharge process constitutes a complete cycle [11].

A model in the context of lithium-ion batteries is a mathematical formulation of the coupled electrochemical, transport, and thermodynamic processes governing cell operation. Most conventional battery models are formulated to describe short-term behavior, defined as the reversible electrochemical response within individual charge–discharge cycles (seconds to hours), including voltage evolution, ionic and electronic transport, and lithium concentration dynamics. Accordingly, several performance-oriented modeling frameworks have been developed, including microscale models, homogenized models, the Doyle–Fuller–Newman (DFN) model, single-particle models with electrolyte dynamics (SPMe), and the single-particle model (SPM) [11].

In contrast, long-term behavior refers to the irreversible evolution of the battery state over extended operation (months to years or hundreds to thousands of cycles), driven by degradation mechanisms such as solid electrolyte interphase growth, lithium plating, particle fracture, and loss of active material. Therefore, a physics-based degradation model is a mathematical and computational framework that describes battery aging by explicitly resolving the physical, chemical, and mechanical processes responsible for irreversible performance loss [13].

The development of a physics-based degradation model follows a systematic methodology. First, the dominant degradation mechanisms are identified based on the operating conditions of interest. Governing equations are then formulated using conservation laws for mass, charge, energy, and momentum, and degradation kinetics are introduced through physically motivated source terms [14]. These equations are coupled with electrochemical transport models describing lithium diffusion in solid particles, ionic transport in the electrolyte, and electronic conduction in the electrodes. The model is closed through appropriate initial and boundary conditions and validated against experimentally observed degradation indicators such as capacity fade and resistance growth [15].

Despite the diversity of degradation mechanisms, physics-based degradation models for lithium-ion batteries share a common mathematical structure rooted in fundamental governing laws. Mass conservation governs the evolution of lithium concentration through a balance between transport and reaction, while flux relations describe lithium transport driven by concentration or chemical potential gradients. Charge conservation ensures current continuity within the solid and electrolyte phases, and electrochemical reaction kinetics are typically formulated using Butler–Volmer-type expressions. Thermal effects are captured through energy conservation, accounting for irreversible heat generation and temperature-dependent degradation rates, while mechanical equilibrium governs stress evolution associated with lithium insertion and extraction [14,15].

These governing principles are commonly expressed through a set of coupled partial differential equations describing species conservation, charge transport, interfacial reaction kinetics, heat transfer, and, where relevant, mechanical deformation [14,15]. Collectively, these equations constitute the shared mathematical backbone of physics-based degradation models for lithium-ion batteries. The general form of these governing equations is summarized below [15].

The electrolyte phase in a lithium-ion battery contains lithium ions dissolved in the liquid electrolyte. During battery operation, these ions move through the electrolyte due to concentration gradients and electric potential gradients. The conservation of mass for lithium ions in the electrolyte is obtained by applying a species balance over a small differential control volume and is given by:

(1) $$\frac{\partial c_{e}}{\partial t}=-\nabla \cdot N_{e}$$

where $c_{e}$ is the electrolyte concentration and $N_{e}$ is the total molar flux of lithium ions in the electrolyte and is given by:

(2) $$N_{e}=-D_{e}\nabla c_{e}-\frac{t_{+}^{0}c_{e}}{F}\nabla \phi_{e}$$

**Figure 2:**
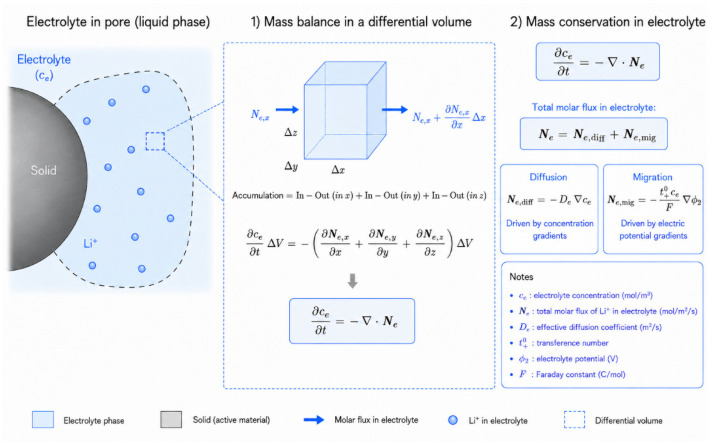
Schematic representation of Li^+^ transport in the electrolyte phase and derivation of the electrolyte mass conservation equation from a differential volume balance.

Where $D_{e}$ is the diffusion of lithium-ions in electrolyte, $t_{+}^{0}$ is transference number of lithium ion, F is Faraday’s constant, $\phi_{e}$ is electrolyte potential (Figure 2).

The conservation of lithium-ions within solid active material particles is expressed as a diffusion equation derived from mass balance [15]:

(3) $$\frac{\partial c_{s}}{\partial t}=-\nabla \cdot J_{s}$$

where $c_{s}$ denotes the lithium concentration in the solid phase and $J_{s}$ is the lithium flux.

**Figure 3:**
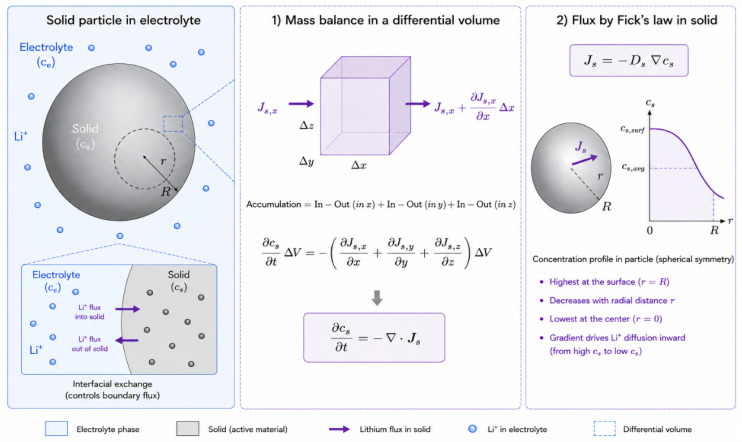
Schematic of solid-phase lithium diffusion and derivation of the governing mass conservation equation in an active-material particle.

The flux is described using Fick’s law [15],

(4) $$J_{s}=-D_{s}\nabla c_{s}$$

where $D_{s}$ is the solid-phase diffusivity. In more general thermodynamic formulations, this flux may be expressed in terms of gradients of chemical potential (Figure 3).

**Figure 4:**
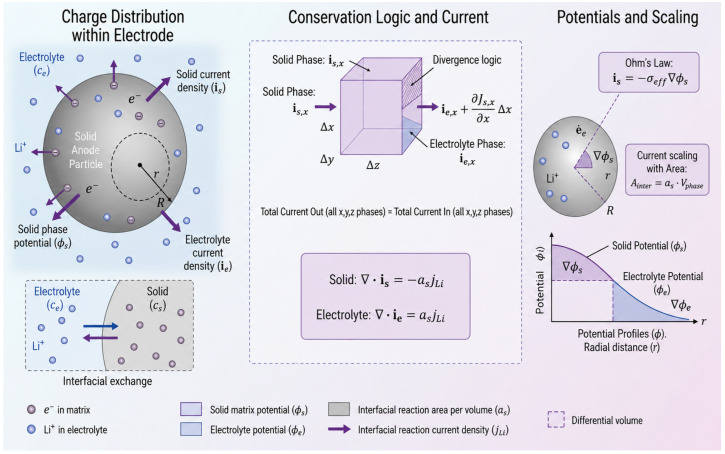
Schematic of charge conservation and potential distribution in a porous electrode, illustrating coupled electronic and ionic current transport between the solid and electrolyte phases.

Charge conservation in the solid and electrolyte phases ensures current continuity and is written as [15]

(5) $$\nabla \cdot i_{s}=-a_{s}j$$

(6) $$\nabla \cdot i_{e}=a_{s}j$$

where $i_{s}$ and $i_{e}$ are the solid and electrolyte-phase current densities, respectively, $a_{s}$ is the specific interfacial area, and j is the interfacial reaction current density (Figure 4).

**Figure 5:**
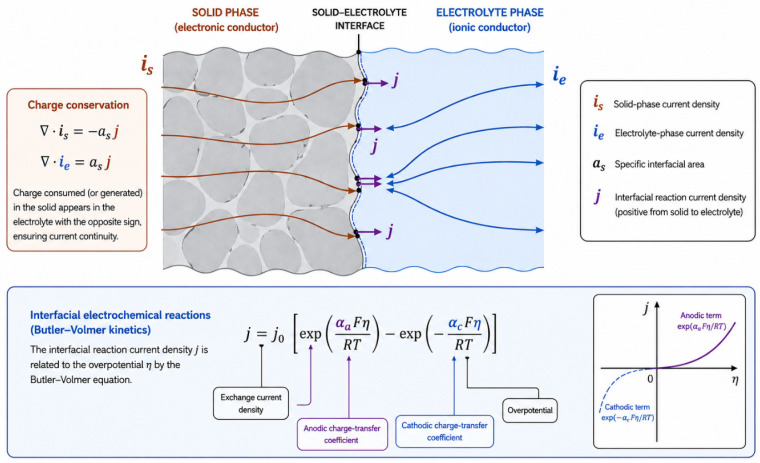
Schematic of interfacial charge transfer between the solid and electrolyte phases, showing charge conservation and Butler–Volmer reaction kinetics at the solid–electrolyte interface.

Interfacial electrochemical reactions are commonly described using Butler–Volmer kinetics [15],

(7) $$j=j_{0}\left[exp\left(\frac{\alpha_{a}F\eta }{RT}\right)-exp\left(\frac{\alpha_{c}F\eta }{RT}\right)\right]$$

where $j_{0}\;$ is the exchange current density, $\alpha_{a}$ and $\alpha_{c}$ are charge-transfer coefficients, and η is the overpotential. In battery modeling, this expression is modified to include additional reaction pathways associated with side reactions such as SEI formation or lithium plating (Figure 5).

**Figure 6:**
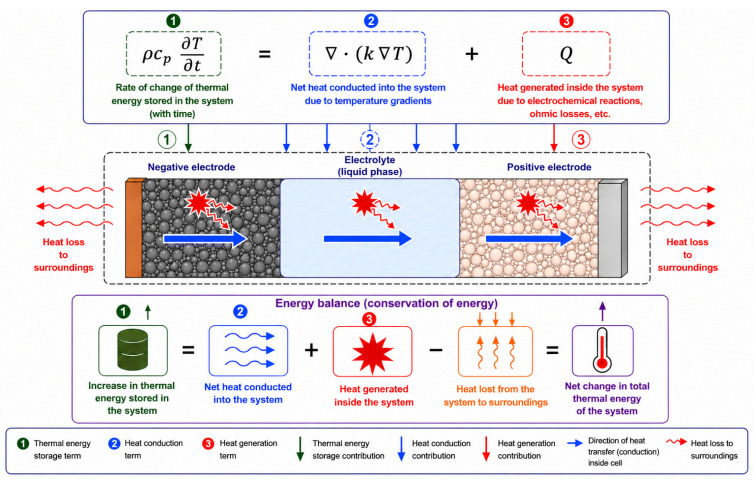
Schematic of thermal energy conservation in a lithium-ion battery, illustrating heat generation, heat conduction, and heat loss mechanisms governed by the energy balance equation. 1, 2, 3 represent different components of the battery cell.

Thermal effects, which strongly influence both electrochemical performance and degradation rates, are incorporated through an energy balance equation [15],

(8) $$\rho c_{p}\frac{\partial T}{\partial t}=\nabla \cdot (k\nabla T)+Q$$

where T is temperature, t is time, ρ is density, $c_{p}$ is the specific heat capacity, k is the thermal conductivity, and Q denotes the volumetric heat generation term (Figure 6).

**Figure 7:**
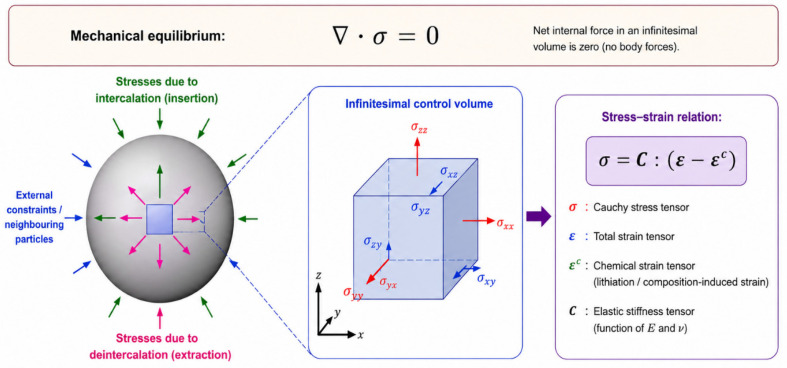
Schematic diagram of stress evolution in an electrode particle during lithium intercalation and deintercalation.

In the context of particle fracture, stress evolution is governed by mechanical equilibrium [15,16],

(9) ∇⋅σ=0

where σ is the Cauchy stress tensor. The stress–strain relation is written as $\sigma =C:(\varepsilon -\varepsilon^{c})$, with ε the total strain tensor, $\varepsilon^{c}$ the chemical strain, and C the elastic stiffness tensor defined by Young’s modulus E and Poisson’s ratio ν (Figure 7).

Together, these equations constitute the core physics-based battery model as presented in [11,14,15].

While empirical degradation models provide approximate lifespan estimates, physics-based degradation models seek to mechanistically link aging phenomena to underlying electrochemical and transport processes under realistic operating conditions [11,15]. Consequently, accurate lifetime prediction requires modeling frameworks capable of coupling short-term electrochemical dynamics with long-term degradation processes. The following section discusses the principal degradation mechanisms affecting lithium-ion batteries.

## 3. Battery Degradation Mechanisms

Degradation mechanisms in lithium-ion batteries refer to the irreversible physicochemical processes that progressively affect the internal state of the cell during operation and storage, leading to deviations from ideal reversible behavior [17,18]. These mechanisms can be divided into two main categories: chemical and mechanical. Chemical degradation is caused by chemical reactions that occur within the battery, such as the decomposition of the electrolyte, the formation of solid electrolyte interphase (SEI) and cathode electrolyte interphase (CEI) layer, lithium plating, dissolution of transition metal, corrosion of current collectors, and gas evolution. This can lead to a loss of capacity, increased impedance, and reduced cycle life.

Physical degradation is caused by physical changes to the battery, such as the formation of cracks in the electrodes, separator breakdown, and loss of active material. This can also lead to a loss of capacity, increased impedance, and reduced cycle life. Both chemical and physical degradation are accelerated by factors such as high temperature, overcharging, and deep discharge [6].

The loss of capacity due to degradation mechanisms is a major limiting factor for the performance of lithium-ion batteries. The following section presents an overview of different degradation mechanisms present in battery literature and explains how particular mechanisms lead to a loss in the capacity of the battery.

### 3.1. Solid Electrolyte Interphase (SEI)

**Figure 8:**
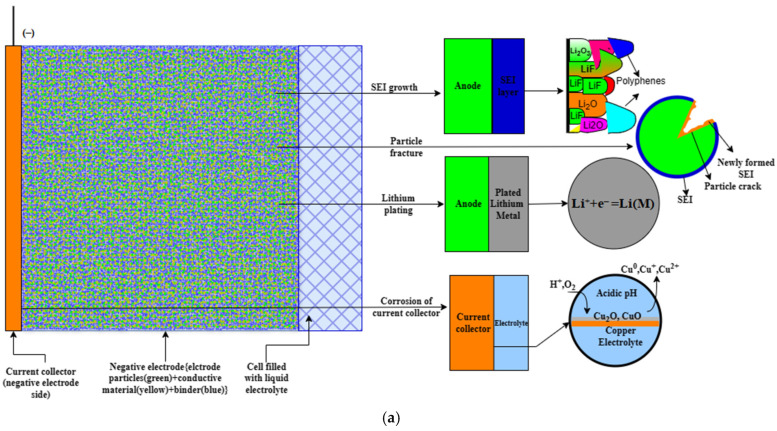

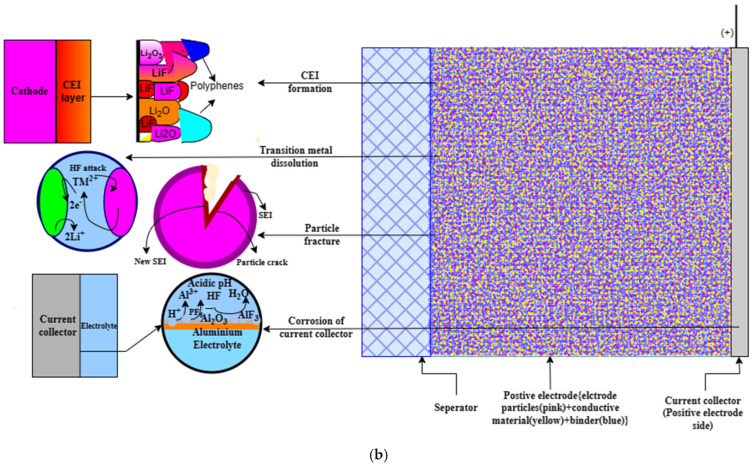
Degradation mechanisms [33] at (**a**) negative electrode (**b**) positive electrode.

Solid electrolyte interphase (SEI) is a protective layer that forms on the surface of the negative electrode during the first charging cycle of a lithium-ion battery. Figure 8a illustrates the formation of the solid electrolyte interphase (SEI) model at the negative electrode. During charging, lithium ions (${\mathrm{Li}}^{+}$) migrate from the cathode through the electrolyte and reach the anode surface, where they combine with electrons supplied through the external circuit. At the anode–electrolyte interface, solvent molecules such as ethylene carbonate (EC) and ethyl methyl carbonate (EMC) undergo reductive decomposition in the presence of ${\mathrm{Li}}^{+}$ and electrons, forming insoluble SEI compounds like lithium fluoride, lithium carbonate, lithium methyl carbonate, lithium ethylene di-carbonate, and lithium oxide. These compounds are thermodynamically stable against lithium, and they form a barrier that prevents further electrolyte decomposition. This passivation layer grows progressively at the anode surface, allowing lithium-ion transport and blocking further electron transfer [19].

During discharge, lithium ions move back into the electrolyte, but the SEI layer remains largely intact, leading to irreversible lithium consumption and gradual impedance growth. Ideally, the formation of the solid electrolyte interphase (SEI) is a one-time event that occurs during the first charging cycle. This process leads to a 10% loss of battery capacity. However, research has shown that the SEI layer can continue to grow over time, even when the battery is not in use. This is because the SEI layer is not as permeable to lithium ions as the electrolyte. This increased impedance can lead to a decrease in battery performance [9,20].

Several coupled electrochemical, thermal, and mechanical factors govern the growth of the solid electrolyte interphase (SEI). Elevated temperatures accelerate parasitic interfacial reactions, leading to increased SEI thickness and altered composition, while high current densities can induce non-uniform lithium fluxes and mechanical stresses that destabilize or fracture the SEI layer. In addition, electrolyte impurities participate in secondary reactions that modify SEI chemistry and transport properties. Owing to the heterogeneous and dynamic nature of the SEI, its formation and evolution remain incompletely understood. Nonetheless, a mechanistic understanding of SEI growth is critical for the development of physics-based models and for improving the reliability and lifetime of lithium-ion batteries [21].

### 3.2. Cathode Electrolyte Interphase (CEI)

The cathode electrolyte interphase (CEI) is a passivation layer that forms on the surface of the positive electrode during the first charging cycle. Figure 8b illustrates the formation of CEI due to the reaction between the positive electrode material and the electrolyte at high voltage. Unlike the SEI, which forms through reductive decomposition of electrolyte at the anode, CEI formation is driven by oxidative reactions of solvent molecules and salt anions under high cathode potentials. These reactions lead to the formation of inorganic and organic species that deposit on the cathode surface.

The CEI layer protects the positive electrode from reacting with the electrolyte, which can cause the battery to short-circuit. This partially passivating layer influences lithium-ion transport and interfacial charge transfer [22]. Excessive CEI growth increases interfacial resistance and contributes to capacity fade and power loss. Moreover, CEI instability can promote transition-metal dissolution from the cathode, which subsequently accelerates anode degradation through cross-linked mechanisms.

Furthermore, the stability of the CEI is highly sensitive to temperature; under elevated thermal conditions, CEI decomposition or restructuring can accelerate parasitic reactions, increase heat generation, and, in extreme cases, trigger thermal runaway. Consequently, accurate representation of CEI evolution is essential for understanding cathode-related degradation and ensuring the safety and durability of lithium-ion batteries.

### 3.3. Lithium Plating

Lithium plating is a degradation phenomenon in lithium-ion batteries that occurs when lithium ions are unable to fully intercalate(insert) into the negative electrode during charging and instead deposit on the electrode surface as metallic lithium [23]. This process can lead to several adverse effects, including capacity loss due to the formation of additional solid electrolyte interphase (SEI) layers from reactions between metallic lithium and the electrolyte, which hinder lithium-ion transport [24].

Figure 8a depicts the lithium plating mechanism, highlighting metallic lithium deposition at the anode–electrolyte interface. Under high charging rates or transport-limited conditions, lithium ions arriving at the anode surface can be reduced directly to metallic lithium, instead of intercalating into the host material. This deposited metallic lithium can form dendritic structures that grow through the separator, increasing the risk of internal short circuits [25,26] and posing serious safety hazards such as thermal runaway, fires, or explosions [27].

The schematic shows lithium plating occurring alongside the SEI layer, with plated lithium accumulating between the SEI and the electrolyte. During discharge, a fraction of the plated lithium may be stripped back into the electrolyte, while the remaining portion becomes electrically isolated dead lithium, contributing to irreversible capacity loss. This model captures the competition between intercalation and plating reactions and their dependence on operating conditions.

Furthermore, The propensity for lithium plating is influenced by multiple factors [23], including high charging rates that favor lithium deposition over intercalation [27], low operating temperatures that reduce lithium diffusivity [28], local microstructural defects that act as nucleation sites for lithium plating [26], elevated charging voltages [13,23], high states of charge [29], and insufficient negative electrode capacity to accommodate incoming lithium during charging [23]. In practice, mitigation strategies include optimizing negative electrode materials, controlling charging protocols, and designing electrodes and separators that suppress dendrite formation and improve tolerance to lithium deposition [30,31,32].

### 3.4. Particle Fracture

Particle fracture is a degradation phenomenon in lithium-ion batteries that arises when active electrode materials are subjected to repeated stress–strain cycles induced by lithium insertion and extraction during charge–discharge operation [34]. Figure 8 illustrates particle fracture induced by diffusion-driven stresses in electrode materials. Lithium intercalation into active material particles results in volumetric expansion, whereas de-lithiation causes contraction. Spatially nonuniform lithium concentration within particles gives rise to internal stress fields, commonly referred to as intercalation-induced stresses, which develop at the particle scale as a consequence of electrochemical transport.

Under repeated charge–discharge cycling, these stresses progressively intensify, leading to crack initiation at the particle surface and subsequent crack propagation toward the particle interior. Particle fracture ultimately results in loss of electrical connectivity, increased reactive surface area, and accelerated electrochemical degradation. The schematic highlights the intrinsic coupling between lithium transport and mechanical fracture processes at the particle level.

Such fracture processes reduce the effective active material available for lithium storage, resulting in capacity fade, and increase internal resistance by disrupting electronic and ionic transport pathways, thereby degrading power capability and energy efficiency. In addition, newly exposed fracture surfaces can promote parasitic reactions with the electrolyte, increasing safety risks and, in severe cases, contributing to thermal instability. Table 1 summarizes the susceptibility of different lithium compositions to volumetric strain and fracture.

It is important to note that volumetric strain induced by lithium insertion and extraction is not the sole contributor to particle fracture in lithium-ion batteries. Additional factors, including the size, morphology, and crystallographic orientation of active material particles such as lithium cobalt oxide, lithium nickel manganese cobalt oxide, and lithium iron phosphate as well as electrolyte composition and manufacturing-induced defects, can significantly influence fracture susceptibility [45]. These factors affect local stress distributions, interfacial stability, and mechanical integrity, thereby accelerating crack initiation and propagation. As particle fracture progresses, the resulting loss of electrical contact, increased interfacial resistance, and enhanced parasitic reactions contribute directly to capacity fade, power loss, and overall battery degradation. Consequently, accounting for these material- and process-dependent effects is essential for accurately modeling mechanical degradation and predicting long-term battery performance.

### 3.5. Dissolution of Transition Metal

Transition metals such as iron in lithium iron phosphate, cobalt in lithium cobalt oxide, and manganese in lithium manganese oxide are essential constituents of many cathode materials, as they provide active sites for electrochemical reactions during battery operation. Under certain operating conditions, however, these transition metals can dissolve into the electrolyte, contributing to several degradation pathways [34,36,38]. Figure 8 illustrates transition metal dissolution in a lithium-ion battery. Transition metal ions are released from the cathode surface due to interfacial chemical instability and enter the electrolyte. These dissolved ions migrate across the electrolyte and deposit at the anode, where they accumulate within the solid electrolyte interphase (SEI) and leads to the loss of electrochemically active material in the cathode, resulting in capacity fade, while dissolved metal ions can participate in parasitic reactions with the electrolyte, producing corrosive species that degrade cell components. In addition, migrated transition metal ions may deposit on the anode or separator, forming electronically conductive pathways that increase internal resistance or, in extreme cases, induce internal short circuits [38].

The dissolution process is governed by multiple factors, including cathode chemistry, with nickel-rich cathodes generally exhibiting higher dissolution rates than lithium iron phosphate systems, as well as operating conditions such as elevated temperature and high charge–discharge rates [36,38]. Electrolyte composition also plays a critical role, as carbonate- and carboxylate-based electrolytes can enhance metal solubility compared to fluorinated solvent systems. To mitigate transition-metal dissolution, several strategies have been proposed, including the selection of cathode materials with lower metal solubility, optimization of operating conditions to limit thermal and electrochemical stress, and the application of protective surface coatings that act as barriers between the cathode and electrolyte [36,40]. Effective control of transition-metal dissolution is therefore essential for minimizing cathode degradation, enhancing safety, and extending the operational lifetime of lithium-ion batteries.

### 3.6. Corrosion of Current Collectors

Corrosion of current collectors is a significant degradation mechanism in lithium-ion batteries, directly affecting electrical integrity, performance, and safety [42,44]. Aluminum and copper, which are commonly used as current collectors for the positive and negative electrodes, respectively, are susceptible to corrosion in the electrochemical environment of the battery. Aluminum current collectors can undergo degradation through galvanic corrosion when coupled with more noble metals, electrochemical dissolution at elevated potentials in the presence of lithium ions and electrolyte species, and stress corrosion cracking induced by residual or operational stresses combined with a corrosive electrolyte environment [44,46,47]. Similarly, copper current collectors are prone to galvanic corrosion when in contact with more noble metals, electrochemical dissolution under unfavorable potential conditions, and environmentally assisted cracking when tensile stress coexists with reactive electrolyte components [46,48,49,50].

Figure 8 depicts the corrosion of current collectors in a lithium-ion battery during electrochemical operation. At the electrolyte interface, unfavorable conditions such as high potential, electrolyte decomposition, or local chemical instability led to oxidation of the metallic current collector. This results in the dissolution of metal species and the formation of corrosion products at the interface. As corrosion progresses, the current collector surface becomes roughened and partially insulated, this leads to a loss of electronic conductivity, increased interfacial resistance, and enhanced parasitic reactions, resulting in capacity fade, increased self-discharge, and, in severe cases, internal short circuits that pose serious safety risks [46]. To mitigate current collector corrosion, various strategies have been proposed, including the application of protective surface coatings, the use of electrolyte additives to improve corrosion resistance, and battery design modifications aimed at reducing mechanical and electrochemical stresses on the collectors [46,48,51,52]. Effective control of current collector corrosion is therefore essential for improving the durability, reliability, and safety of lithium-ion batteries.

## 4. Battery Degradation Models: A Comparative Study

Lithium-ion battery degradation is commonly quantified by the number of charge–discharge cycles sustained before the usable capacity declines to 80% of its initial value; however, additional factors such as aging and impedance growth under rest conditions also play a critical role in performance decline [53]. Incorporating degradation mechanisms such as solid electrolyte interphase growth, lithium plating, and particle fracture into operational battery models requires augmenting the governing equations with additional state variables, source terms, and constitutive relations, including film growth laws, electrochemical plating kinetics, and fracture or damage criteria [11]. On this basis, the existing literature can be systematically organized into distinct classes of degradation models, encompassing foundational formulations, isothermal and non-isothermal growth models, morphology-resolved approaches, and multiscale frameworks. Although these classes differ in their level of physical detail and computational complexity, they all originate from a common set of conservation laws and interfacial kinetics [13]. Each class is discussed in detail in the sections that follow, with emphasis on its assumptions, scope of applicability, and limitations.

### 4.1. SEI Degradation Models

SEI degradation models are developed by extending the operational physics-based battery framework to include interfacial reaction kinetics and film evolution equations that account for electrolyte reduction and passivation at the negative electrode, along with lithium and solvent consumption terms, SEI thickness or resistance growth relations, and modified interfacial overpotentials to capture transport limitations and potential drops across the growing interphase [54,55]. Through this coupling, SEI growth directly influences lithium inventory loss, impedance rise, and overall cell performance.

Depending on the level of physical detail and the manner in which these additional formulations are introduced, SEI degradation models reported in the literature can be broadly categorized into foundational models, SEI growth models, morphology-resolved models, and multiscale models. Each category reflects different assumptions regarding SEI structure, growth kinetics, and coupling across length scales, and is examined in detail in the following section.

#### 4.1.1. Foundational SEI Models

Foundational SEI models establish the conceptual basis for interfacial passivation in lithium-ion batteries and are limited to describing the initial formation of the solid electrolyte interphase at the negative electrode. The seminal work of Peled et al. [56] introduced the SEI as a solid passivation layer formed by electrolyte reduction at the negative electrode, enabling reversible lithium intercalation by blocking electron transport while allowing lithium-ion conduction. This concept fundamentally changed the understanding of electrode–electrolyte stability and laid the groundwork for all subsequent SEI modeling efforts. Peled et al. [56] defines an apparent SEI thickness as:

(10) $$L_{SEI}=\frac{L_{SEI}^{0}}{\theta }$$

(11) $$L_{SEI}^{0}=\frac{\varepsilon \;\varepsilon_{0}\;A}{C_{SEI}^{0}}$$

where ε denotes the dielectric constant of the solid electrolyte interphase (SEI), $\varepsilon_{0}$ indicates the permittivity of free space, A is the electrochemically active electrode surface area, θ is a phenomenological dispersion parameter and $C_{SEI}^{0}$ represents the apparent SEI capacitance obtained from impedance measurements under the assumption of ideal capacitive behavior (θ=1). This expression interprets the SEI as an effective dielectric layer, allowing its thickness to be inferred from measured capacitance values.

Subsequent studies by Peled et al. [57,58] extended this framework by considering SEI formation in liquid and polymer electrolytes and introducing the mosaic structure concept, which emphasized the heterogeneous and multiphase nature of the SEI. Experimental investigations by Fong et al. [59] and Ein-Eli et al. [60] further reinforced these ideas by linking SEI formation to first-cycle irreversible capacity loss and electrical double-layer behavior.

Comprehensive experimental reviews by Aurbach [61] and later by Peled et al. [62] synthesized electrochemical, spectroscopic, and microscopy evidence to establish the now-accepted picture of a layered SEI composed of inorganic inner layers and organic outer layers. Although these foundational models are essential for establishing the physical basis of SEI formation and stability, they do not explicitly capture time-dependent SEI growth, lithium inventory loss, or the resulting capacity fade. Consequently, such models are not predictive degradation models; rather, they provide the underlying conceptual assumptions and physical constraints upon which physics-based degradation modeling frameworks are developed.

#### 4.1.2. Isothermal SEI Growth Models

SEI growth models constitute the first class of quantitative degradation models capable of predicting irreversible lithium consumption and capacity fade. These models explicitly couple interfacial side reactions with transport processes and typically assume that SEI growth is governed by a single dominant rate-limiting mechanism. Early reaction–diffusion formulations by Christensen and Newman [63] described SEI growth as a consequence of solvent diffusion through the SEI, resulting in a square root of time growth law that successfully explained long-term calendar aging trends. This approach was further formalized by Ploehn et al. [64], who derived analytical expressions linking SEI thickness growth to solvent transport and irreversible capacity loss.

Analytical extensions by Pinson et al. [65] unified SEI growth with porous-electrode theory, demonstrating that lithium inventory loss emerges naturally from side reactions without requiring empirical capacity-fade laws. Similar physics-based formulations were proposed by Ramasamy et al. [66] and Sankarasubramanian et al. [67] modeled SEI growth during storage and cycling and highlighted the importance of reaction kinetics at the electrode–SEI interface. More recent studies, such as Reddy et al. [68] and Perassi et al. [69], extended SEI growth models to account for open-circuit aging and evolving active surface area, respectively. Collectively, these SEI growth models form the backbone of physics-based degradation modeling and remain widely used due to their analytical clarity and computational efficiency.

This loss mechanism is introduced through a lithium mass balance in the solid phase augmented by a sink term representing the SEI-forming side reaction [65]

(12) $$\frac{\partial c_{s}}{\partial t}=-\frac{a_{s}}{F}\;j_{SEI}$$

where $c_{s}$ is the lithium concentration in the solid electrode (mol $m^{-3}$), t denotes time (s), $a_{s}$ is the specific interfacial surface area ($m^{2}$/m^3^), F is the Faraday constant (${\mathrm{Cmol}}^{-1}$), and $j_{SEI}$ is the SEI formation current density (${\mathrm{Am}}^{-2}$).

Further this framework is extended by introducing an explicit evolution equation for the interphase thickness [68],

(13) $$\frac{d\delta_{SEI}}{dt}=\frac{M_{SEI}}{\rho_{SEI}\;nF}\;j_{SEI}$$

where $\delta_{SEI}$ is the SEI thickness, $M_{SEI}$ and $\rho_{SEI}$ are the molar mass and density of the SEI, respectively, and n is the number of electrons involved in the SEI reaction. This relation assumes uniform growth normal to the electrode surface and converts the interfacial reaction current into a physical growth rate, enabling prediction of SEI thickness and resistance evolution over time.

Isothermal SEI growth models assume spatially uniform and temporally constant temperature, neglecting thermal gradients and heat generation within the cell. In these formulations, temperature effects are either ignored or implicitly included through Arrhenius-type dependencies of reaction rate constants and diffusion coefficients. Models by Christensen and Newman [63], Ploehn et al. [64], Pinson et al. [65], Ramasamy et al. [66], and Sankarasubramanian et al. [67] fall into this category. Their primary strength lies in their ability to isolate the fundamental electrochemical and transport mechanisms governing SEI growth while maintaining mathematical and computational tractability.

Isothermal electrochemical models relax the assumption of purely transport-limited growth by explicitly resolving reaction kinetics using Butler–Volmer-type expressions [65],

(14) $$j_{SEI}=j_{0,SEI}\left[exp\;\left(\frac{\alpha F\eta }{RT}\right)-\exp\;\left(-\frac{\left(1-\alpha \right)F\eta }{RT}\right)\right]$$

where $j_{0,SEI}$ is the exchange current density, α is the charge-transfer coefficient, η is the overpotential, R is the universal gas constant, and T is temperature. This formulation introduces explicit dependence of SEI growth on local electrochemical driving forces, enabling models to capture the influence of state of charge and applied current. Temperature effects are commonly incorporated through Arrhenius-type relations, $k=k_{0}exp(-E_{a}/RT)$, accounting for the thermally activated nature of SEI-forming reactions even under nominally isothermal conditions.

However, isothermal models inherently neglect the strong coupling between degradation and temperature, which becomes significant under high-rate cycling, elevated ambient temperatures, or large-format cell operation. As a result, while isothermal formulations are well suited for calendar aging studies and low-rate cycling, their predictive accuracy diminishes under realistic operating conditions where thermal effects are non-negligible.

#### 4.1.3. Non-Isothermal SEI Growth Models

Non-isothermal SEI growth models explicitly account for the coupling between electrochemical degradation and thermal transport by solving energy conservation equations alongside mass and charge balance. A study by Liu et al. [70] demonstrated that SEI growth kinetics are strongly temperature dependent and that even modest temperature increases can significantly accelerate irreversible lithium loss. System-level investigations by Lawder et al. [71] incorporated non-isothermal SEI growth into porous-electrode models subjected to realistic drive cycles, revealing complex feedback between resistance growth, heat generation, and degradation rate.

More recent degradation frameworks, such as those proposed by Zhang et al. [72] and Zhou et al. [73], further emphasized the importance of thermal effects when multiple degradation mechanisms coexist. While non-isothermal models offer improved realism and applicability to high-power operation, they require additional thermal parameters and incur greater computational cost. Nevertheless, they are essential for predicting degradation behavior under fast charging, high-current operation, and thermal abuse conditions.

While standard cycling gradually thickens the SEI, extreme operating conditions accelerate structural failure. Zhou et al. [74] studied NCM batteries under high-rate discharge (up to 10 C) and overdischarge (down to 0.5 V). By applying a thermal energy balance and using Bragg’s Law to track lattice spacing, they demonstrated that extreme temperatures reaching 134.78 °C trigger repeated SEI/CEI decomposition and transition metal migration, leading to irreversible structural collapse.

#### 4.1.4. Morphology-Resolved SEI Models

Morphology-resolved SEI models represent a significant advancement beyond spatially averaged growth formulations by explicitly resolving SEI heterogeneity, porosity evolution, and layered structure. Initial work by Single et al. [75] introduced spatially resolved SEI models that predicted the emergence of porous and compact SEI regions as a natural consequence of competing transport and reaction processes. Subsequent theoretical analysis by Single et al. [76] clarified the physical origins of SEI morphology and linked SEI structure to electrochemically observable quantities.

Morphology-resolved SEI models extend these approaches by accounting for the evolving microstructure of the interphase and its impact on transport properties by introducing an effective diffusivity of the form [75,76]:

(15) $$D_{eff}=\varepsilon^{\;b}D_{bulk}$$

where ε represents SEI porosity, b is a morphology-dependent exponent related to tortuosity, and $D_{bulk}$ is the intrinsic diffusivity of the species within the SEI material. Such formulations recognize that SEI morphology actively governs long-term growth kinetics rather than being a passive consequence of degradation.

Further studies by Single et al. [77,78], demonstrated that classical solvent-diffusion-limited models cannot simultaneously explain time and state-of-charge dependence observed experimentally. This conclusion was further reinforced by Köbbing et al. [79] and Kolzenberg et al. [80,81] who showed that electron or lithium-interstitial diffusion mechanisms provide a consistent explanation for long-term SEI growth. While morphology-resolved models offer mechanistic insight into the SEI growth, their computational complexity and parameter sensitivity pose challenges for large-scale lifetime prediction.

#### 4.1.5. Multiscale SEI Models

Parameter uncertainty refers to the lack of precise, reliable values for key model parameters that govern SEI growth, such as reaction rate constants, transport coefficients, activation energies, electronic conductivity, and material properties of SEI. In traditional SEI growth models, these parameters are often treated as fixed constants, even though they depend strongly on local conditions (temperature, state of charge, electrolyte composition) and evolve as the SEI itself changes in structure and chemistry over time. As a result, different parameter choices can lead to widely varying predictions of SEI thickness, growth rate, and capacity fade, limiting the predictive capability of such models.

Multiscale SEI models reduce this uncertainty by linking continuum-scale parameters to atomistic or mesoscale calculations (e.g., density functional theory or molecular dynamics), where reaction energetics and transport mechanisms are explicitly resolved. This hierarchical coupling provides a physically grounded basis for parameter selection, improving model robustness and transferability across operating conditions. First-principles studies by Shi et al. [82] revealed lithium-interstitial diffusion mechanisms within inorganic SEI components, providing a physical basis for transport assumptions used in continuum models. Complementary density-functional-theory investigations by Peng et al. [83] presented electrolyte decomposition pathways and activation energies governing SEI formation.

Molecular dynamics and Monte Carlo studies, such as those by Takenaka et al. [84], further enriched understanding of SEI composition and transport behavior. Comprehensive reviews by Horstmann et al. [77] synthesized these multiscale efforts and emphasized the necessity of linking quantum chemistry, mesoscale transport, and continuum degradation modeling. In summary, multiscale SEI models play a crucial enabling role in modern degradation modeling by providing physically grounded parameters and mechanistic insight that cannot be obtained from continuum-scale formulations alone. Although these approaches do not typically predict capacity fade directly, they significantly reduce parameter uncertainty and offer systematically derived validation of reaction pathways, transport mechanisms, and interfacial evolution, thereby strengthening the foundations of higher-level predictive models.

The evolution of SEI degradation modeling reflects a progression from early conceptual passivation theories to physics-based kinetic descriptions, morphology-resolved formulations, and multiscale frameworks. Each modeling class captures specific aspects of SEI behavior and operates within a limited range of assumptions and length scales; consequently, no single approach is sufficient in isolation to describe the full complexity of SEI-driven degradation. Accurate lifetime prediction, therefore, requires a hierarchical, integrated modeling strategy that consistently links atomistic chemistry, mesoscale morphology, and continuum-scale electrochemical performance, motivating the development of unified frameworks that couple electrochemical, thermal, mechanical, and chemical degradation processes within a single theoretical structure. Table 2 summarizes the key physical processes, advantages, and limitations of the different SEI modeling approaches discussed in this section.

### 4.2. Lithium Plating Degradation Models

Lithium plating degradation models are theoretical and computational frameworks developed to describe the electrochemical deposition of metallic lithium on the negative electrode of lithium-ion batteries and to assess its impact on performance degradation and safety. Similar to SEI degradation, lithium plating modeling has evolved through distinct stages of theoretical development, and in this review, such models are classified using the same conceptual framework as SEI models to enable systematic comparison across degradation mechanisms.

#### 4.2.1. Foundational Lithium Plating Models

Foundational lithium plating models establish the fundamental electrochemical and transport conditions under which metallic lithium deposition occurs. The seminal work of Arora et al. [85] introduced lithium plating as a side reaction within the Doyle–Fuller–Newman porous electrode framework, describing lithium deposition using Butler–Volmer kinetics activated when the local negative-electrode potential falls below the lithium metal equilibrium potential. This formulation provided the first rigorous physics-based criterion for lithium plating onset and demonstrated that plating is fundamentally a transport-induced electrochemical instability rather than a purely material failure.

The electrochemical rate of lithium plating is described using Butler–Volmer-type kinetics. Arora et al. [85] expressed the plating current density as

(16) $$j_{\mathrm{pl}}=j_{0,\mathrm{pl}}\left[exp\;\left(\frac{\alpha F\eta_{\mathrm{pl}}}{RT}\right)-\exp\;\left(-\frac{\left(1-\alpha \right)F\eta_{\mathrm{pl}}}{RT}\right)\right]$$

where $j_{0,\mathrm{pl}}$ is the exchange current density of the plating reaction, α is the charge-transfer coefficient, $\eta_{\mathrm{pl}}$ is the lithium plating overpotential, R is the universal gas constant, and T is the absolute temperature. During charging, the stripping term is often neglected, resulting in a net plating current.

Complementary theoretical insight was provided by Monroe et al. [86] performing a classical stability analysis for lithium metal growth based on surface energy and mechanical constraints. Although originally formulated for lithium-metal batteries, this theory forms the mechanistic basis for understanding dendritic growth following lithium plating in lithium-ion systems. Together, these foundational studies provide clear physical explanation and onset criteria but do not capture plating morphology, reversibility, or long-term capacity degradation.

#### 4.2.2. Isothermal Lithium Plating Growth Models

Lithium plating growth models extend foundational formulations by explicitly describing the rate and temporal evolution of metallic lithium deposition through coupled electrochemical kinetics and transport processes. In these models, lithium plating is treated as a competing interfacial reaction to lithium intercalation, typically activated when the local electrode potential falls below the equilibrium potential of metallic lithium. The deposition rate is commonly described using Butler–Volmer-type kinetics, linking the plating current density to overpotential, temperature, and exchange current density (Arora et al. [85]).

To translate interfacial kinetics into physical accumulation, growth models introduce evolution equations for plated lithium thickness or volume fraction, converting the plating current into a geometric growth rate [87]. These formulations capture the nonlinear nature of lithium plating, including rapid growth under fast charging and partial reversibility during discharge. To account for irreversible capacity loss, many studies incorporate a stripping efficiency or dead-lithium fraction, allowing a portion of plated lithium to become electrochemically inactive over repeated cycles [31]. Alyea et al. [88] proposed a more advanced growth model to further couple plating kinetics with electrolyte transport and interfacial resistance, improving predictions under high-rate and low-temperature conditions.

To relate the electrochemical plating rate to physical lithium accumulation, growth models introduce a plated-lithium thickness evolution equation. Yang et al. [31] expressed this relation as

(17) $$\frac{d\delta_{\mathrm{pl}}}{dt}=\frac{M_{\mathrm{Li}}}{\rho_{\mathrm{Li}}F}\;j_{\mathrm{pl}}$$

where $\delta_{\mathrm{pl}}$ is the thickness of the plated lithium layer, $M_{\mathrm{Li}}$ is the molar mass of lithium, and $\rho_{\mathrm{Li}}$ is the density of metallic lithium. This equation enables direct prediction of lithium buildup, interfacial resistance growth, and capacity loss. Electrolyte transport enters lithium plating models through the definition of the plating overpotential. Yang et al. [31] formulated the overpotential as

(18) $$\eta_{\mathrm{pl}}=\phi_{s}-\phi_{e}-U_{{\mathrm{Li}/\mathrm{Li}}^{+}}-\frac{RT}{F}ln(a_{{\mathrm{Li}}^{+}})$$

Here, $\phi_{s}$ and $\phi_{e}$ are the electric potentials in the solid and electrolyte phases, respectively, $U_{{\mathrm{Li}/\mathrm{Li}}^{+}}$ is the equilibrium potential of the lithium metal reaction, and $a_{{\mathrm{Li}}^{+}}$ is the activity of lithium ions in the electrolyte. This formulation shows that lithium plating naturally arises under transport-limited conditions.

Isothermal lithium plating models resolve lithium deposition kinetics under the assumption of spatially uniform and constant temperature, allowing electrochemical and transport effects to be isolated from thermal coupling. In these models, lithium plating is typically described using Butler–Volmer-type kinetics, with the plating current density expressed as a function of local overpotential and electrolyte concentration, while temperature-dependent parameters are treated as constants evaluated at a reference temperature [85]. Isothermal formulations have been widely used to identify plating onset conditions during fast charging and low-temperature operation, demonstrating that localized electrolyte depletion and potential drops can trigger lithium deposition even when average electrode potentials remain above the lithium equilibrium potential [31].

Although these models do not capture thermal feedback effects, they provide a computationally efficient and physically transparent framework for analyzing electrochemical and transport-driven lithium plating behavior and serve as a foundation for more advanced non-isothermal and multiscale approaches.

#### 4.2.3. Non-Isothermal Lithium Plating Growth Models

Non-isothermal lithium plating models extend isothermal formulations by explicitly resolving temperature evolution and its coupling with electrochemical kinetics, transport processes, and interfacial resistance. In these models, the plating current density retains a Butler–Volmer-type form, but kinetic parameters such as exchange current density, electrolyte conductivity, and diffusion coefficients are treated as temperature-dependent, commonly through Arrhenius relations. Temperature fields are obtained from an energy conservation equation accounting for reversible heat, ohmic losses, and reaction heat, enabling capture of feedback mechanisms between heat generation, local overpotential, and lithium deposition and is governed by [89]:

(19) $$\rho C_{p}\frac{\partial T}{\partial t}=\nabla \cdot (k\nabla T)+Q_{\mathrm{rxn}}+Q_{\mathrm{ohmic}}$$

where ρ is the density of the medium, $C_{p}$ is the specific heat capacity, k is the thermal conductivity, and $Q_{\mathrm{rxn}}$ and $Q_{\mathrm{ohmic}}$ denote heat generation due to electrochemical reactions and ohmic losses, respectively. These studies demonstrated that spatial temperature gradients alone can induce localized lithium plating even when average voltage indicators suggest safe operation.

Temperature dependence of lithium plating kinetics is commonly introduced through Arrhenius-type relations. Ren et al. [28] expressed the temperature dependence of the plating exchange current density as

(20) $$j_{0,\mathrm{pl}}=j_{0,\mathrm{pl},\mathrm{ref}}\exp\left(-\frac{E_{a}}{R}\left(\frac{1}{T}-\frac{1}{T_{\mathrm{ref}}}\right)\right)$$

where $j_{0,\mathrm{pl},\mathrm{ref}}$ is the reference exchange current density at temperature $T_{\mathrm{ref}}$, and $E_{a}$ is the activation energy for the plating reaction.

Non-isothermal models have demonstrated that local temperature gradients can significantly accelerate lithium plating by enhancing reaction kinetics while simultaneously increasing transport polarization, particularly under fast-charging conditions [90,91,92]. By coupling thermal and electrochemical effects, these models provide improved predictive capability for lithium plating onset, growth rate, and associated safety risks compared to isothermal approaches, and form a necessary bridge toward morphology-resolved and multiscale plating models [93].

The progression of degradation mechanisms like lithium plating significantly increases the risk of thermal runaway. Liu et al. [94] quantified this fire risk as a product of fire probability (P) and consequence severity. Their modeling revealed that while fire probability scales linearly with the state of charge (SOC), the highest consequence severity occurs at 80% SOC due to specific gas accumulation. Furthermore, in stable chemistries like LFP, Liu et al. [95] utilized a transient thermal balance model to show that electric-thermal coupling (simultaneous heating and short-circuiting) generates temperatures exceeding 1000 °C, causing intense combustion that would not occur under single-source abuse.

Electrochemical–thermal models parameterized using operando NMR and electrochemical measurements by Ren et al. [28] demonstrated that lithium plating can occur even at relatively low states of charge under cold conditions due to suppressed lithium-ion transport. More advanced three-dimensional thermo-electrochemical simulations by Vishnugopi et al. [96] and Sun et al. [97] further revealed that spatial temperature gradients alone can induce highly inhomogeneous lithium plating, even when the average cell voltage remains within nominal limits. These findings highlight a critical limitation of voltage-based diagnostics and underscore that lithium plating risk cannot be reliably assessed without accounting for coupled thermal effects. Although non-isothermal models significantly improve predictive realism, they require additional thermal parameters and incur higher computational cost.

#### 4.2.4. Morphology-Resolved Lithium Plating Models

Morphology-resolved lithium plating models advance beyond spatially averaged porous-electrode descriptions by explicitly resolving the spatial distribution, connectivity, and structural evolution of plated lithium. Three-dimensional microstructure-resolved simulations introduced by Hein [98,99] demonstrated that lithium deposition is highly localized, arising from microscopic current density amplification within heterogeneous anode microstructures. Building on this framework, Fang et al. [100] incorporated explicit tracking of plated lithium thickness and its associated electronic resistance in realistic graphite anodes, revealing strong feedback between local transport limitations and plating growth.

Morphology-resolved lithium plating models explicitly capture the spatial non-uniformity of lithium deposition arising from local current-density amplification within heterogeneous electrode microstructures. Hein and Latz [99] expressed the local plating rate as

(21) $$j_{\mathrm{pl}}(x)=j_{0,\mathrm{pl}}exp\left(\frac{F\eta_{\mathrm{pl}}(x)}{RT}\right)$$

where $j_{\mathrm{pl}}(x)$ is the spatially varying plating current density and $\eta_{\mathrm{pl}}(x)$ is the local plating overpotential. This formulation shows that lithium deposition can become highly localized even under moderate average charging conditions. Fang et al. [100] further resolved the local growth of plated lithium thickness using

(22) $$\frac{\partial \delta_{\mathrm{pl}}(x)}{\partial t}=\frac{M_{\mathrm{Li}}}{\rho_{\mathrm{Li}}F}\;j_{\mathrm{pl}}(x)$$

where $\delta_{\mathrm{pl}}(x)$ denotes the local plated lithium thickness.

More recently, Sahu et al. [25] proposed a continuum formulation that distinguishes reversible plated lithium, electrically connected dendritic lithium, and electrochemically inactive dead lithium, enabling quantitative prediction of plating reversibility and irreversible capacity loss. Sahu et al. [25] introduced separate state variables for reversibly plated lithium, electrically connected dendrites, and inactive dead lithium, enabling quantitative prediction of plating reversibility and irreversible capacity loss.

Collectively, these models demonstrate that lithium plating morphology is not merely a geometric consequence but an active regulator of degradation kinetics and safety risk. Nevertheless, their high computational expense and extensive microstructural parameter requirements currently restrict their applicability to full-cell lifetime prediction and long-term degradation assessment.

#### 4.2.5. Multiscale Lithium Plating Models

Multiscale lithium plating models integrate electrochemical, thermal, mechanical, and thermodynamic phenomena across particle, electrode, and cell scales, providing a unified description of plating initiation and evolution under realistic operating conditions. Based on phase-field and porous-electrode assumptions, Thomas-Alyea et al. [88] demonstrated that graphite staging and phase coexistence can generate highly localized regions of elevated lithium chemical potential, thereby triggering lithium plating even when the spatially averaged electrode potential remains above the lithium metal equilibrium potential. These results established that lithium plating is fundamentally coupled to bulk intercalation thermodynamics and phase behavior, rather than being governed solely by surface overpotential criteria.

Thomas-Alyea et al. [88] modified lithium transport equation by coupling porous-electrode theory and phase-field thermodynamics is given by

(23) $$\frac{\partial c_{s}}{\partial t}=\nabla \cdot \left(M\nabla \mu \right)$$

where $c_{s}$ is the solid-phase lithium concentration, M is the mobility coefficient of lithium in the solid phase. and μ is the chemical potential. This approach showed that phase coexistence can generate local thermodynamic driving forces for lithium plating even when macroscopic electrode potentials appear safe.

Building on this perspective, electro-chemo-mechanical models developed by Zhang et al. [93] incorporated stress evolution, contact mechanics, and pressure effects, revealing that mechanical constraints can redirect lithium deposition pathways and promote localized plating instead of uniformly suppressing it. Zhang et al. [93] equation for modified chemical potential is

(24) $$\mu =\mu_{0}+RTlna+\Omega \sigma_{h}$$

where μ is the lithium chemical potential under mechanical loading, $\mu_{0}$ is the reference chemical potential, a is the activity of lithium in the host material, R is the universal gas constant, T is the absolute temperature, Ω is the partial molar volume of lithium, and $\sigma_{h}$ is the hydrostatic stress. This expression accounts for the coupling between electrochemical thermodynamics and mechanical stress in lithium plating.

This demonstrates that stress can redirect lithium deposition pathways rather than simply suppressing plating.

In parallel, studies by O’Kane et al. [13] showed that voltage signatures commonly attributed to lithium plating can also arise from graphite phase transitions in the absence of metallic deposition, highlighting fundamental ambiguities in voltage-based detection methods. They found that the diffusion coefficient of lithium in the active electrode material can have a significant impact on the rate of lithium plating and stripping. Although multiscale lithium plating models offer the most physically comprehensive framework available, their high parameterization requirements and substantial computational cost limit their use primarily to mechanistic insight and benchmark studies, rather than routine long-term lifetime prediction.

Overall, lithium plating degradation models have evolved in a way similar to SEI degradation models. Early studies focused on identifying when lithium plating starts, while later models described how plated lithium grows, how its shape and distribution change, and how plating is affected by processes occurring across particle, electrode, and cell scales. Unlike SEI growth, which mainly causes a slow increase in resistance, lithium plating can lead to sudden loss of usable lithium and poses a higher safety risk. Because of this complexity, no single modeling approach can describe all aspects of lithium plating behavior. Accurate prediction therefore requires combining models at different scales and consistently accounting for electrochemical reactions, transport limitations, morphology changes, and thermal effects, along with careful interpretation of experimental signals. Table 3 presents a comparison of the main lithium plating degradation models, summarizing their key physical processes, advantages, and limitations.

In summary, lithium plating models differ primarily in their level of physical detail and spatial resolution, ranging from simplified continuum descriptions to morphology-resolved and multiscale formulations. The key distinctions lie in how these models represent plating reversibility, microstructural evolution of deposited lithium, thermal effects, and interactions across multiple length scales. Rather than reflecting fundamentally different modeling philosophies, these variations reflect increasing efforts to capture the complex, coupled nature of lithium plating under realistic operating conditions. This viewpoint enables lithium plating models to be consistently integrated into broader battery degradation and lifetime prediction frameworks.

### 4.3. Particle Fracture Degradation Models in Lithium-Ion Batteries

Particle fracture models describe mechanical degradation of electrode particles caused by stresses generated during battery operation. Unlike purely electrochemical degradation mechanisms, these models explicitly couple lithium transport with mechanical deformation to capture stress development and damage evolution. Based on their modeling scope and level of coupling, the existing literature is commonly classified into foundational stress models for electrode particles, diffusion-induced stress models with chemo-mechanical coupling, fracture-mechanics-based particle failure models, and multiscale chemo-mechanical fracture models. Each class differs in how mechanical stresses are formulated, how they are coupled with lithium transport, and how damage or failure is represented. The following sections systematically describe these model classes, emphasizing their underlying assumptions and distinguishing modeling features.

#### 4.3.1. Foundational Stress Models for Electrode Particles

The earliest modeling efforts addressing mechanical degradation in lithium-ion batteries focused on quantifying stress generation within electrode particles during lithiation and de-lithiation, without explicitly resolving crack initiation or propagation. These foundational models established the fundamental link between lithium concentration gradients and internal stresses. Christensen [103] developed one of the earliest continuum descriptions of diffusion-induced stress in spherical electrode particles by coupling Fickian lithium diffusion with linear elasticity, demonstrating that nonuniform lithiation leads to tensile and compressive stress fields [104].

The transport of lithium ions within an individual particle is commonly described using Fick’s second law [103],

(25) $$\frac{\partial c}{\partial t}=\nabla \cdot (D\nabla c)$$

where c is the lithium concentration in the solid particle and D is the solid-state lithium diffusivity. Stress formulations developed by Christensen [103,104].and Yang [105] also follow the same assumption and provide the first quantitative link between charging rate, particle size, and stress generation.

Subsequent analytical studies further clarified that even in the absence of external constraints, internal stresses arise naturally due to concentration-dependent volumetric expansion. Yang et al. [105] and Wu et al. [106] showed that stress magnitudes increase sharply at high C-rates and large particle radii, suggesting a mechanical origin for capacity loss observed experimentally. A major drawback of these foundational stress models is that they remained limited to elastic deformation and did not include material failure criteria, making them insufficient for predicting fracture-induced degradation directly.

#### 4.3.2. Diffusion-Induced Stress Models with Chemo-Mechanical Coupling

To overcome the limitations of purely elastic formulations, later models incorporated explicit chemo-mechanical coupling by allowing stress to influence lithium transport and vice versa. Deshpande et al. [89] introduced a thermodynamically consistent framework in which lithium diffusion was driven by gradients in chemical potential rather than concentration alone, thereby incorporating stress effects into transport kinetics. This approach demonstrated that compressive and tensile stresses can significantly retard or accelerate lithium diffusion, altering both stress evolution and lithiation uniformity.

To convert lithium concentration changes into mechanical deformation, diffusion-induced stress models introduce a constitutive relation between concentration and strain, typically expressed through a Vegard-type relation [103]

(26) $$\varepsilon_{\mathrm{chem}}=\beta (c-c_{0})$$

where $\varepsilon_{\mathrm{chem}}$ is the chemically induced strain, β is the partial molar expansion coefficient, and $c_{0}$ is a reference lithium concentration. When coupled with linear elasticity, this formulation yields stress fields that depend on material stiffness, particle geometry, and cycling conditions. Christensen [103] used this framework to show that tensile hoop stresses develop near particle surfaces during fast lithiation, providing a mechanical explanation for surface crack initiation.

Christensen [107] extended these ideas by considering nonlinear elastic behavior and showed that stress-assisted diffusion can lead to stress localization near particle surfaces. Ai et al. [108] and Li et al. [109] further refined chemo-mechanical models by incorporating concentration-dependent elastic moduli and anisotropic deformation, particularly relevant for high-volume-change materials such as silicon.

More advanced chemo-mechanical models account for feedback between stress and lithium transport by expressing diffusion in terms of chemical potential rather than concentration alone. Deshpande et al. [110] formulated lithium flux as

(27) $$J=-\frac{Dc}{RT}\nabla \mu$$

where J is the lithium flux, R is the universal gas constant, T is temperature, and μ is the chemical potential, which includes both chemical and mechanical contributions. In this framework, stress alters lithium diffusion kinetics, while evolving concentration fields further modify stress distributions.

Christensen [107], as well as Ai et al. [108] and Li et al. [109], showed that stress-assisted diffusion can localize lithium near particle surfaces, intensifying stress concentrations, an effect particularly important for high-volume-change materials such as silicon. While these diffusion-induced stress models provided a more realistic description of coupled electrochemical–mechanical behavior, fracture was not modeled explicitly and was instead inferred when computed stresses exceeded prescribed strength criteria.

#### 4.3.3. Fracture-Mechanics-Based Particle Failure Models

A major conceptual advance in particle fracture modeling was achieved by explicitly introducing fracture mechanics principles into electrode degradation analysis. Woodford et al. [111] developed the concept of electrochemical shock, in which diffusion-induced stresses drive crack initiation and unstable crack growth in brittle electrode materials. By combining stress solutions with Griffith-type fracture criteria, they derived critical C-rates above which particle fracture becomes inevitable and introduced electrochemical shock maps that relate particle size, charging rate, and fracture toughness.

To explicitly predict crack initiation, fracture-mechanics-based models incorporate failure criteria grounded in fracture mechanics. Woodford et al. [111] introduced the concept of electrochemical shock by coupling diffusion-induced stress solutions with Griffith’s fracture criterion [111],

(28) $$G\geq G_{c}$$

where G is the elastic energy release rate and $G_{c}$ is the critical fracture energy of the material. This approach enabled prediction of critical particle sizes and charging rates beyond which fracture becomes inevitable. Subsequent finite-element studies by Zhang et al. [16] explicitly resolved crack initiation and propagation, revealing strong sensitivity to particle geometry, pre-existing flaws, and cycling protocols.

Building on this framework, Zhu et al. [34] developed finite-element models that explicitly resolved crack initiation and propagation in primary particles under lithiation-induced stress. Their work demonstrated that crack evolution depends not only on peak stress but also on stress gradients and loading history. Zhao et al. [112] and Zhang et al. [16] further extended fracture-mechanics-based models to high-capacity electrode materials, showing that fracture onset strongly correlates with particle morphology, pre-existing flaws, and cycling protocols.

More recently, damage-mechanics and phase-field fracture models have been developed to capture progressive fracture over repeated cycles. In these approaches, a continuously evolving damage or phase-field variable represents gradual stiffness degradation and crack growth, avoiding the need to predefine crack paths. Zhu et al. [34] and Liu et al. [113] demonstrated that phase-field models can naturally capture crack nucleation, branching, and interaction, making them particularly suitable for simulating fatigue-driven particle degradation and loss of active material connectivity.

Battery modeling based on fracture-mechanics represents a critical step toward quantitatively linking mechanical failure to electrochemical degradation but often require detailed material parameters that are difficult to measure experimentally, but their practical application is often limited by the need for material parameters that are difficult to measure experimentally. The next section focuses on multiscale chemo-mechanical fracture models, which extend these formulations by linking particle-scale damage to electrode- and cell-level behavior.

#### 4.3.4. Multiscale Chemo-Mechanical Fracture Models

Recent modeling efforts have moved toward multiscale frameworks that integrate particle-level fracture with electrode- and cell-level behavior. These models recognize that particle fracture not only reduces active material connectivity but also accelerates secondary degradation processes such as SEI reformation and progressive lithium consumption. Bower et al. [114] and Cui et al. [115] introduced multiscale approaches in which particle-scale stress and fracture feed back into electrode-scale transport properties through evolving porosity and surface area.

To explicitly model crack initiation and growth within a multiscale framework, phase-field fracture formulations are frequently employed. Lee et al. [116] and Zhu et al. [34,117] gave the total free-energy functional as:

(29) $$E=\int_{\Omega }\left[g(d)\;\psi_{\mathrm{el}}(\varepsilon )+G_{c}\left(\frac{d^{2}}{2l}+\frac{l}{2}{|\nabla d|}^{2}\right)\right]d\Omega$$

where d is the damage (phase-field) variable, $\psi_{\mathrm{el}}$ is the elastic strain-energy density, $G_{c}$ is the fracture toughness, and l is the regularization length scale. This approach enables prediction of crack nucleation, growth, and interaction without prescribing crack paths, and has been applied to battery particles by Zhu et al. [34] and Liu et al. [113].

At the electrode scale, particle fracture is coupled to transport and connectivity by degrading effective material properties. For example, Sengupta et al. [118] and Zhang et al. [119] related particle-scale damage to loss of electronic connectivity through

(30) $$\sigma_{s}^{\mathrm{eff}}=\sigma_{s}(1-d)$$

where $\sigma_{s}^{\mathrm{eff}}$ is the effective solid-phase conductivity and d represents fracture-induced damage. This coupling links particle cracking to electrode-scale resistance growth and capacity fade.

Liu et al. [113] and Sengupta et al. [118] employed phase-field and damage-mechanics formulations to simulate progressive fracture across multiple cycles, enabling prediction of gradual stiffness loss and capacity fade. Zhang et al. [119] further demonstrated that stress-controlled charging strategies derived from particle-scale fracture models can significantly mitigate mechanical degradation, highlighting the direct link between modeling and operational optimization. While multiscale fracture models offer the most physically comprehensive description of particle degradation, their computational cost and parameter complexity currently limit their widespread use in lifetime prediction and battery management applications.

Overall, particle fracture models have evolved from simplified stress estimations to fully coupled, multiscale chemo-mechanical frameworks capable of resolving crack initiation, propagation, and degradation feedback. Foundational stress models provide analytical clarity but lack failure prediction; diffusion-induced stress models capture coupling effects but treat fracture implicitly; fracture-mechanics-based models enable quantitative failure criteria; and multiscale models integrate mechanical degradation into long-term performance prediction.

Despite significant progress, a unified framework that seamlessly couples fracture, SEI evolution, lithium plating, and thermal effects across scales remains an open challenge, underscoring the need for integrated degradation modeling strategies in next-generation lithium-ion batteries. Table 4 compares the major classes of particle fracture models, highlighting their dominant physical mechanisms, key assumptions, modeling scale, advantages, and limitations.

In summary, particle fracture models share a common mathematical foundation built on lithium diffusion, chemically induced strain, mechanical equilibrium, and failure criteria. Differences between model classes arise from the degree of chemo-mechanical coupling, whether fracture is treated implicitly or explicitly, and the extent of scale integration. While foundational stress models offer analytical insight, fracture-mechanics-based and multiscale approaches provide greater predictive capability at the cost of increased complexity, underscoring the need for hierarchical integration within comprehensive battery degradation frameworks.

To provide a broader theoretical context for this hierarchical scaling, it is essential to link these localized particle-level phenomena to the macroscopic framework of Continuum Damage Mechanics (CDM). Within CDM, material degradation is quantified by an empirical damage variable, D, ranging from an undamaged state (D = 0) to a state of complete failure (D = 1). Historically, researchers have conceptualized this damage variable through various empirical surrogates. For instance, the Palmgren-Miner hypothesis [120] calculates damage as a linear accumulation of load cycle fractions, while subsequent models linked damage to measurable microstructural changes. These include Lemaitre’s [121] and Voyiadjis’ [122] stiffness degradation [122], Kachanov’s [123] reduction in effective load-bearing area, and Rousselier’s [124] void density framework.

The practical application of these macro-level CDM principles to cell-level degradation is evident in recent advancements in Lithium-ion battery (LIB) characterization, which bridge particle-level damage mechanisms with bulk structural failure. For example, Voyiadjis et al. [125] operationalized the concept of stiffness degradation by developing an inverse optimization procedure to extract elastoplastic stress-strain relationships directly from indentation data. Building on this, they introduced a time-fractional derivative (Caputo-Almeida) into a CDM framework to capture the memory and rate-dependent effects of structural softening [126]. Furthermore, Akbari and Voyiadjis [127,128] demonstrated how these damage variables can be integrated into VUMAT subroutines to predict the onset of internal short circuits (ISCs) across various form factors and loading regimes.

**Figure 9:**
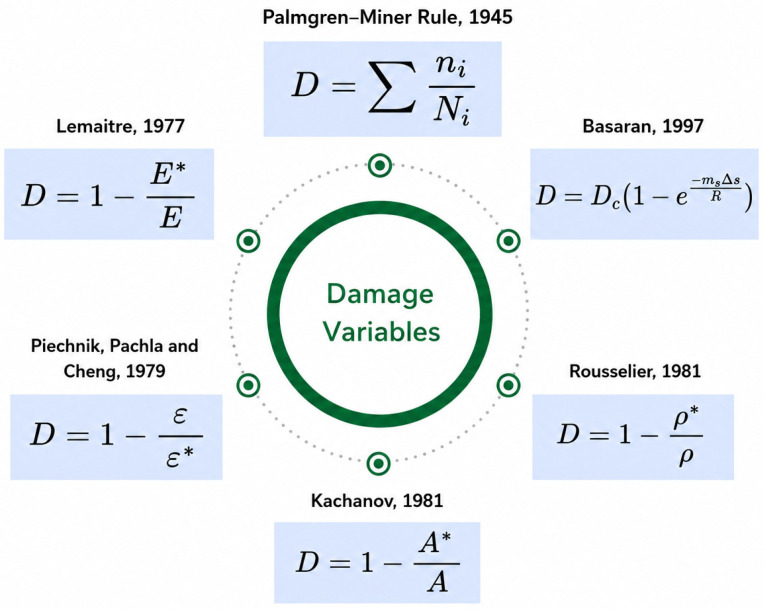
Schematic diagram showing different damage variables proposed in continuum damage mechanics theories. Adapted from Palmgren–Miner (1945) [120], Lemaitre (1977) [121], Piechnik, Pachla and Cheng (1979) [122], Kachanov (1981) [123], Rousselier (1981) [124], and Basaran (1997) [129]. Superscript * denotes degraded value.

This transition from localized particle fracture and mechanical characterization to a more holistic physics-based predictive framework provides the necessary foundation for the thermodynamic unification proposed in this work. The Unified Mechanics Theory (UMT), proposed by Cemal Basaran in 1997, introduces entropy as the fundamental measure of damage [129]. As illustrated in Figure 9, Basaran uses the Thermodynamic State Index (TSI) as the degradation variable, which is defined as a function of entropy generation mechanisms in UMT. Because entropy is a scalar quantity, it can be universally summed across varying and multifaceted loading conditions—such as the electrical, thermal, and mechanical stresses inherent in battery cycling—to determine the system’s overall health. This approach provides a robust, thermodynamically consistent framework for predicting the onset and progression of failure, and the specific mathematical details of this formulation are presented in the following section.

## 5. Entropy-Based Degradation Modeling

A fundamental limitation of conventional degradation modeling approaches lies in their reliance on mechanism-specific state variables to quantify degradation, such as SEI thickness, plated lithium volume, or crack density, which are not easily comparable across degradation modes. Moreover, these models typically assess degradation in terms of observable outcomes (capacity fade, resistance growth, fracture onset) rather than through a common thermodynamic measure of irreversibility. This fragmentation complicates the development of unified lifetime prediction frameworks and limits the direct transferability of insights across materials, cell formats, and operating conditions. In this context, entropy-based degradation frameworks, such as those proposed within the Unified Mechanics Theory (UMT) [129], offer a fundamentally different modeling approach.

The Unified Mechanics Theory (UMT) is an ab initio unification of the laws of Newton and the second law of thermodynamics, as formulated by Boltzmann and Planck [130]. As a result, UMT introduces a fifth linearly independent axis, the Thermodynamic State Index (TSI), into the Newtonian space-time coordinate system. TSI is the normalized form of the second law of thermodynamics. TSI coordinates range from zero to one. When the entropy is zero at the initial reference state, TSI is zero; when the entropy is near maximum, TSI asymptotically approaches one. UMT enables thermodynamics-based description of degradation by capturing the coupled electrochemical, thermal, and mechanical phenomena that drive battery aging. In UMT, lifetime degradation occurs according to the second law of thermodynamics, as formulated by Boltzmann and Planck [130]. As such, the fundamental thermodynamic equation that describes entropy generation mechanisms must be derived.

Unified Mechanics Theory has been applied to a variety of engineering problems involving progressive damage and failure driven by irreversible processes. The framework has been primarily developed and validated in the context of solid mechanics, including fatigue damage and fracture in metals and composite materials, thermo-mechanical degradation of structural components, solder joints in electronic packaging, hydrogen embrittlement, etc. [42,129,130,131,132,133,134,135,136,137,138,139]. In these applications, damage evolution is formulated in terms of entropy generation based on second law, providing thermodynamically consistent degradation laws that have shown good agreement with experimental observations and improved robustness compared to conventional phenomenological damage models.

For electrochemical energy storage systems, irreversible entropy production is associated with charge-transfer overpotentials, ohmic losses, concentration gradients, which makes it a natural measure of cumulative degradation.

In recent years, entropy has been increasingly explored as a degradation metric in battery modeling. Early phenomenological studies by Cuadras et al. [140] demonstrated that irreversible entropy generation, computed from experimentally measured voltage, current, and temperature, correlates strongly with capacity and power fade in rechargeable batteries. Subsequent work by Cuadras et al. [141] extended this idea to operational optimization, showing that charging and discharging strategies minimizing entropy production also reduce thermal stress and improve energy efficiency, particularly under aging and vehicle-to-grid operating condition.

When interpreted through an entropy-based approach, SEI growth corresponds to irreversible chemical entropy generation due to side reactions and transport resistance, lithium plating contributes through electrochemical irreversibility and loss of usable free energy, and particle fracture manifests as mechanical dissipation and irreversible structural damage. Importantly, these contributions can be accumulated consistently over time and operating cycles, enabling the definition of a thermodynamic state index that evolves monotonically toward failure. Such a framework naturally accommodates coupled electrochemical–mechanical–thermal effects, thereby offering a complementary alternative to highly detailed physics-based models.

While standard literature predominantly investigates isolated degradation phenomena, a critical and largely unaddressed domain in battery prognostics involves modeling battery aging as a combined, synergistic system. To date, comprehensive numerical frameworks that explicitly account for the simultaneous confluence of multiple distinct aging mechanisms remain exceptionally scarce. A seminal exception is the multi-mechanism framework developed by O’Kane et al. [13] within a Doyle-Fuller-Newman (DFN) electrochemical model. This architecture directly couples four foundational degradative pathways localized at the negative electrode: solid electrolyte interphase (SEI) growth, lithium plating, active particle fracture, and the loss of active material (LAM). Their findings demonstrate that concomitant degradation mechanisms are inherently non-linear and coupled rather than merely additive. Specifically, the authors mathematically captured the regulatory effect of SEI layer thickness on the passivation kinetics of plated lithium into “dead lithium,” while concurrently demonstrating how mechanical particle fracture accelerates chemical SEI growth by continually exposing pristine electrode surfaces to the electrolyte. By mapping these interfacial coupled dynamics, they established that macroscale battery capacity fade and end-of-life trajectories are highly path-dependent, governed strictly by the operational temperature and charging protocol.

However, the fact that this highly coupled, physics-based approach stands virtually alone in literature highlights a significant research gap. Explicitly tracking, parameterizing, and validating every permutation of mechanical, chemical, and electrical cross-interaction introduces severe mathematical complexity and computational overhead, which has historically hindered the realization of a fully unified degradation framework. The scarcity of such multi-mechanism formulations underscores the necessity of transitioning toward a universal thermodynamic paradigm. Integrating high-fidelity mechanistic insights, such as those introduced by O’Kane et al. [13], with entropy-based methodologies like the Unified Mechanics Theory (UMT) represents a highly promising pathway to overcome these limitations. By bridging localized, multi-physics phenomena with a thermodynamically consistent scalar damage accumulation variable, such hybrid frameworks offer a viable solution to the mathematical challenges of multi-mechanism degradation modeling, thereby significantly advancing predictive lifetime modeling for next-generation energy storage systems.

## 6. Conclusions

The transition toward sustainable energy systems and the widespread adoption of electric vehicles (EVs) are fundamentally dependent on the predictive accuracy of lithium-ion battery degradation models. These models serve as critical tools for optimizing battery design, evaluating safety risks, and developing operational strategies such as stress-controlled charging protocols that extend the functional lifespan of energy storage systems. This review has examined physics-based modeling approaches for three dominant degradation mechanisms: solid electrolyte interphase (SEI) growth, lithium plating, and particle fracture. Although these mechanisms are often modeled separately, they share a common physical foundation based on mass, charge, and energy conservation, and are therefore strongly coupled during real battery operation.

A key insight of this review is that battery degradation is rarely the result of isolated phenomena; rather, it is driven by complex interconnections between chemical, physical, and thermal modes. For example, particle fracture does not merely reduce capacity through loss of active material; it also creates newly exposed surfaces that accelerate secondary parasitic reactions, such as the reformation of the Solid Electrolyte Interphase (SEI). Similarly, lithium plating can trigger the growth of additional SEI layers, while transition metal dissolution from the cathode can migrate through the electrolyte to deposit on the anode, further destabilising the SEI and increasing internal resistance. These “cross-linked mechanisms” are often amplified by thermal feedback loops, where degradation-induced heat accelerates reaction kinetics, which in turn causes further degradation.

Current modeling trends reflect a necessary shift from describing individual components to capturing these coupled multiphysics interactions. While isothermal growth models provide computational efficiency for standard aging, they are insufficient for extreme operational conditions such as fast charging or cold-temperature operation, where non-isothermal and morphology-resolved models are required to track localized lithium deposition and mechanical stress. These limitations underscore the need for a unified degradation framework. By formulating degradation in terms of irreversible entropy generation, Unified Mechanics Theory (UMT) offers a thermodynamically consistent approach that integrates mechanical, chemical, and electrochemical dissipation into a single, monotonic measure of battery health.

Ultimately, the future of battery modeling lies in integrated degradation strategies that link atomistic insights with continuum-scale performance. By bridging mechanistic understanding with unified frameworks, researchers can achieve the predictive robustness necessary to safely extend the operational limits of batteries in next-generation transportation and grid-scale energy storage application.

## Figures and Tables

**Table 1:** List of electrode materials that are most susceptible to particle fracture.

Electrode Material	Change in Volume (% Lithium Insertion/Exertion)
Graphite [35,36]	~10
Silicon [37,38]	200–300
Lithium titanate [39,40]	<3
Lithium iron phosphate [41,42]	<6
Lithium cobalt oxide [43,44]	~6

**Table 2:** Classification and Comparison of SEI Degradation Models.

Model Class	Representative References	Dominant Physics	Temperature Treatment	Advantages	Limitations
Foundational SEI models	Peled et al. [56,57,58]; Fong et al. [59]; Ein-Eli et al. [60]; Aurbach et al. [61]	Passivation concept, selective transport	Not applicable	Establishes physical basis of SEI; conceptually robust	No kinetics, no capacity-fade prediction
SEI growth models (Isothermal)	Christensen et al. [63]; Ploehn et al. [64]; Pinson et al. [65]; Ramasamy et al. [66]; Sankarasubramanian et al. [67]; Reddy et al. [68]; Perassi et al. [69];	Reaction–diffusion, transport-limited growth	Isothermal	Analytical clarity; computationally efficient	Neglects thermal effects; uniform SEI assumption
SEI growth models (Non-isothermal)	Liu et al. [70]; Lawder et al. [71]; Zhang et al. [72] and Zhou et al. [73]	Electrochemical–thermal coupling	Non-isothermal	Captures temperature-accelerated degradation	Higher complexity; additional parameters
Morphology-resolved SEI models	Single et al. [75,76,77,78]; Köbbing et al. [79]; Kolzenberg et al. [80,81]	Spatial heterogeneity; porosity evolution	Mostly isothermal	Mechanism discrimination; struct prediction	High computational cost
Multiscale SEI models	Shi et al. [82]; Peng et al. [83]; Takenaka et al. [84]; Horstmann et al. [77]	DFT/MD + continuum coupling	Implicit via parameters	Physically grounded parameters	Indirect lifetime prediction

**Table 3:** Classification and Comparison of Lithium Plating Degradation Models.

Model Class	Representative References	Dominant Physics	Temperature Treatment	Advantages	Limitations
Foundational lithium plating models	Arora et al. [85]; Monroe [86]	Electrochemical side reaction; stability of Li metal growth	Implicit/Isothermal	Clear physical onset criteria; mechanistic interpretation	No morphology resolution; empirical lifetime prediction
Lithium plating growth models (Isothermal)	Yang et al. [31,101]; Perkins et al. [102]; Hein et al. [98]	Butler–Volmer kinetics; transport-limited overpotential	Isothermal	Captures reversibility and voltage signatures; computationally efficient	Neglects thermal gradients; spatial homogeneity assumed
Lithium plating growth models (Non-isothermal)	Ge et al. [91]; Ren et al. [28]; Zhao et al. [90]; Petzl et al. [92]; Zhang et al. [93]; Vishnugopi et al. [96]; Sun et al. [97]	Electrochemical–thermal coupling; temperature-dependent kinetics	Non-isothermal	Realistic fast-charging and cold-temperature prediction	Higher complexity; thermal parameters required
Morphology-resolved lithium plating models	Hein et a. [98,99]; Fang et al. [100]; Sahu et al. [25]; Wood et al. [26]	Spatially resolved deposition; dendrite and dead-lithium formation	Mostly isothermal	Predicts localization, morphology, and safety risk	High computational cost; limited scalability
Multiscale lithium plating models	Thomas-Alyea et al. [88]; Zhang et al. [93]; O’Kane et al. [13]	Phase-field thermodynamics; electro-chemo-mechanical coupling	Implicit/ Partially coupled	Physically comprehensive; diagnostic interpretation	Parameter-intensive; indirect lifetime prediction

**Table 4:** Classification and Comparison of Particle Fracture Modeling Approaches.

Model Class	Representative References	Dominant Physics	Temperature Treatment	Advantages	Limitations
Foundational stress models	Christensen et al. [103,104]; Yang et al. [105]; Wu et al. [106];	Diffusion-induced stress due to concentration gradients; linear elasticity	Isothermal	Establishes fundamental link between lithiation and stress generation; analytical clarity	No explicit fracture prediction; elastic assumption; cannot quantify capacity loss
Diffusion-induced stress models with chemo-mechanical coupling	Deshpande et al. [89,110]; Christensen et al. [107]; Ai et al. [108] and Li et al. [109]	Coupled lithium diffusion and stress via chemical potential; nonlinear elasticity	Mostly isothermal	Captures feedback between stress and transport; applicable to high-volume-change materials	Fracture treated implicitly; requires material parameters often difficult to measure
Fracture-mechanics-based particle failure models	Woodford et al. [111]; Zhao et al. [112]; Zhang et al. [16]; Zhu et al. [34]	Griffith-type fracture criteria; electrochemical shock; crack initiation and propagation	Isothermal	Quantitative prediction of fracture onset; links C-rate, particle size, and toughness	Computationally intensive; sensitive to flaw size and fracture toughness
Multiscale chemo-mechanical fracture models	Zhu et al. [34,117]; Bower et al. [114]; Cui et al. [115]; Lee [116]; Sengupta et al. [118]; Liu et al. [113]; Zhang et al. [119]	Particle-scale fracture coupled to electrode-scale transport and degradation	Implicit or partially coupled	Integrates fracture with capacity fade, SEI growth, and performance loss	High parameter complexity; limited suitability for real-time lifetime prediction

## Data Availability

No new data were created or analyzed in this study.
